# Evidence for interannual persistence of infectious influenza A viruses in Alaska wetlands

**DOI:** 10.1016/j.scitotenv.2021.150078

**Published:** 2021-09-03

**Authors:** Andrew M. Ramey, Andrew B. Reeves, Benjamin J. Lagassé, Vijay Patil, Laura E. Hubbard, Dana W. Kolpin, R. Blaine McCleskey, Deborah A. Repert, David E. Stallknecht, Rebecca L. Poulson

**Affiliations:** a U. S. Geological Survey, Alaska Science Center, 4210 University Drive, Anchorage, AK 99508, USA; b Department of Biology and Wildlife, University of Alaska Fairbanks, P.O. Box 756100, Fairbanks, AK 99775, USA; c U. S. Geological Survey, Upper Midwest Water Science Center, 8505 Research Way, Middleton, WI 53562, USA; d U. S. Geological Survey, Central Midwest Water Science Center, 400 S. Clinton Street, Iowa City, IA 52244, USA; e U. S. Geological Survey, Water Resources Mission Area, 3215 Marine Street, Building 6, Boulder, CO 80309, USA; f Southeastern Cooperative Wildlife Disease Study, Department of Population Health, College of Veterinary Medicine, University of Georgia, Athens, GA 30602, USA

**Keywords:** Alaska, Avian, Bird, Environment, Flu, Persistence, Reservoir

## Abstract

Influenza A viruses (IAVs) deposited by wild birds into the environment may lead to sporadic mortality events and economically costly outbreaks among domestic birds. There is a paucity of information, however, regarding the persistence of infectious IAVs within the environment following deposition. In this investigation, we assessed the persistence of 12 IAVs that were present in cloacal and/or oropharyngeal swabs of naturally infected ducks. Infectivity of these IAVs was monitored over approximately one year with each virus tested in five water types: (1) distilled water held in the lab at 4 °C and (2–5) filtered surface water from each of four Alaska sites and maintained in the field at ambient temperature. By evaluating infectivity of IAVs *in ovo* following sample retrieval at four successive time points, we observed declines in IAV infectivity through time. Many viruses persisted for extended periods, as evidenced by ≥25% of IAVs remaining infectious in replicate samples for each treatment type through three sampling time points (144–155 days post-sample collection) and two viruses remaining viable in a single replicate sample each when tested upon collection at a fourth time point (361–377 days post-sample collection). The estimated probability of persistence of infectious IAVs in all five water types was estimated to be between 0.25 and 0.75 during days 50–200 post-sample collection as inferred through Kaplan-Meier survival analysis. Our results provide evidence that IAVs may remain infectious for extended periods, up to or even exceeding one year, when maintained in surface waters under ambient temperatures. Therefore, wetlands may represent an important medium in which infectious IAVs may reside outside of a biotic reservoir.

## Graphical Abstract

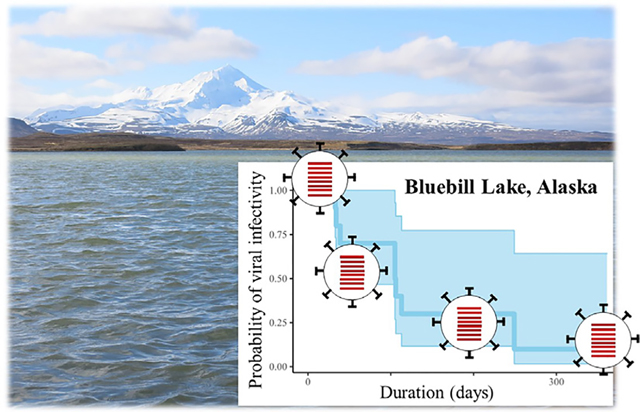

## 1. Introduction

Waterfowl, gulls, and shorebirds play an important role in the maintenance and dispersal of diverse influenza A viruses (IAVs), including those of the H5 and H7 subtypes which may lead to economically costly poultry outbreaks (Swayne, 2008; Bevins et al., 2016). Though IAVs are presumed to be transmitted among wild birds primarily through environmental surface waters, many previous research and surveillance efforts have prioritized sampling of aquatic birds for IAVs, rather than the wetland habitats they occupy, resulting in important data gaps (Coombe et al., 2021). For example, relatively few investigations have reported the isolation of IAVs directly from naturally occurring wetland surface waters and the recovery of viable viruses have been limited to times and locations when waterfowl and gulls have been present or had only recently departed (Hinshaw et al., 1979; Sivanandan et al., 1991; Ito et al., 1995; Stallknecht et al., 2010; Lebarbenchon et al., 2011; Okuya et al., 2015). Even fewer investigations have reported the isolation of IAVs from sediments collected from coastal or wetland habitats recently used by wild birds (Poulson et al., 2017). Thus, information is currently limited regarding the persistence of infectious IAVs within the environment following deposition by wild bird hosts. Additional information on the duration of infectivity for IAVs in the environment would, therefore, be useful for better understanding the mechanisms governing the maintenance of viruses within the wild bird reservoir. Such information may also help to inform the development and implementation of management actions to detect and mitigate viral spread during outbreaks of highly pathogenic clade 2.3.4.4 Goose/Guangdong lineage H5 influenza in wild birds, as have recently occurred in Africa, Asia, Europe, and North America (Saito et al., 2015; Bevins et al., 2016; Lycett et al., 2016; Abolnik et al., 2019; Lycett et al., 2020).

Until recently, information on the duration of IAV infectivity in water has been largely based upon temperature, pH, and salinity-controlled experiments conducted in laboratory settings using laboratory propagated virus (Stallknecht et al., 1990a; Zarkov, 2006; Brown et al., 2007; Brown et al., 2009; Domanska-Blicharz et al., 2010; Keeler et al., 2014; Zhang et al., 2014; Dalziel et al., 2016). More recently, two field experiments were conducted to explore the persistence of infectious IAVs present in the feces or on tissues of naturally infected ducks when maintained in filtered surface water under ambient temperatures in North American wetlands (Reeves et al., 2020; Ramey et al., 2020). Generally, both laboratory- and field-based approaches have provided consistent evidence that IAVs may remain infectious for extended periods (e.g., exceeding six months) when maintained in cold water (approaching 0 °C), with near-neutral pH (approaching 7.0), and exhibiting low to moderate salinity/specific conductivity (fresh to brackish water).

Given evidence for the influence of physical and chemical factors on the persistence of infectious IAVs within water and the recent development of field-based approaches to assess the duration of viral infectivity, we aimed to evaluate viral persistence within waterfowl habitats exhibiting variable physical and chemical properties at a local scale. We focused our field efforts at Izembek National Wildlife Refuge (NWR), Alaska, a location globally recognized for its importance as wetland habitat to hundreds of thousands of migratory waterfowl (U.S. Fish and Wildlife Service, 1986; Ramsar Sites Information Service, 2020), a well-established sampling site for avian-origin IAVs in the northwestern United States of America (Reeves et al., 2018), and a purported point of entry for viruses from East Asia into North America (Ramey et al., 2018).

## 2. Materials and methods

### 2.1. Establishment of field sites, pre-experiment preparation, and initial characterization of surface waters

**Fig. 1.**
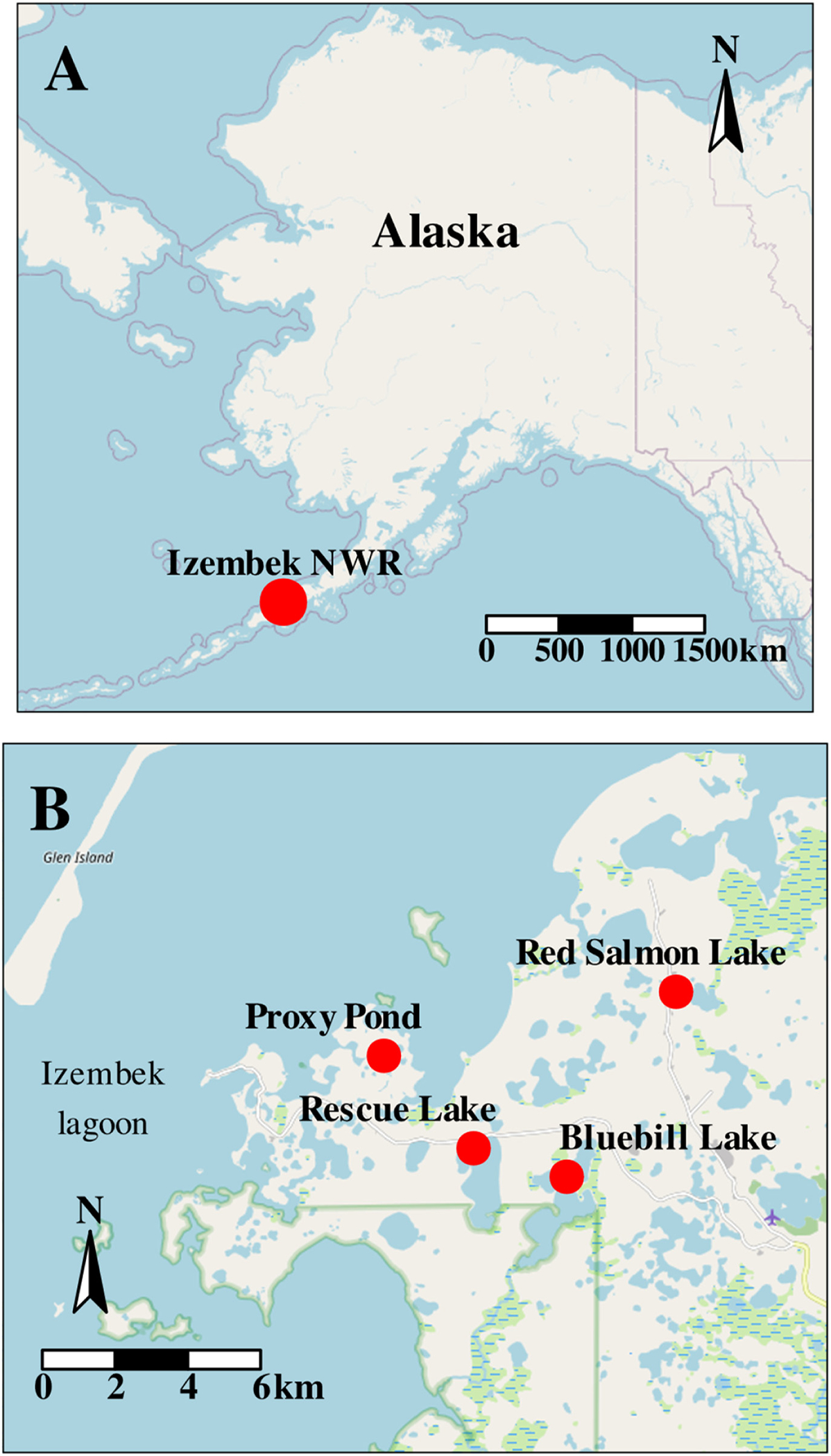
Map of Alaska depicting the approximate location of Izembek National Wildlife Refuge (red circle, Panel A) and the relative position of four field sites therewithin (red circles, Panel B).

**Fig. 2.**
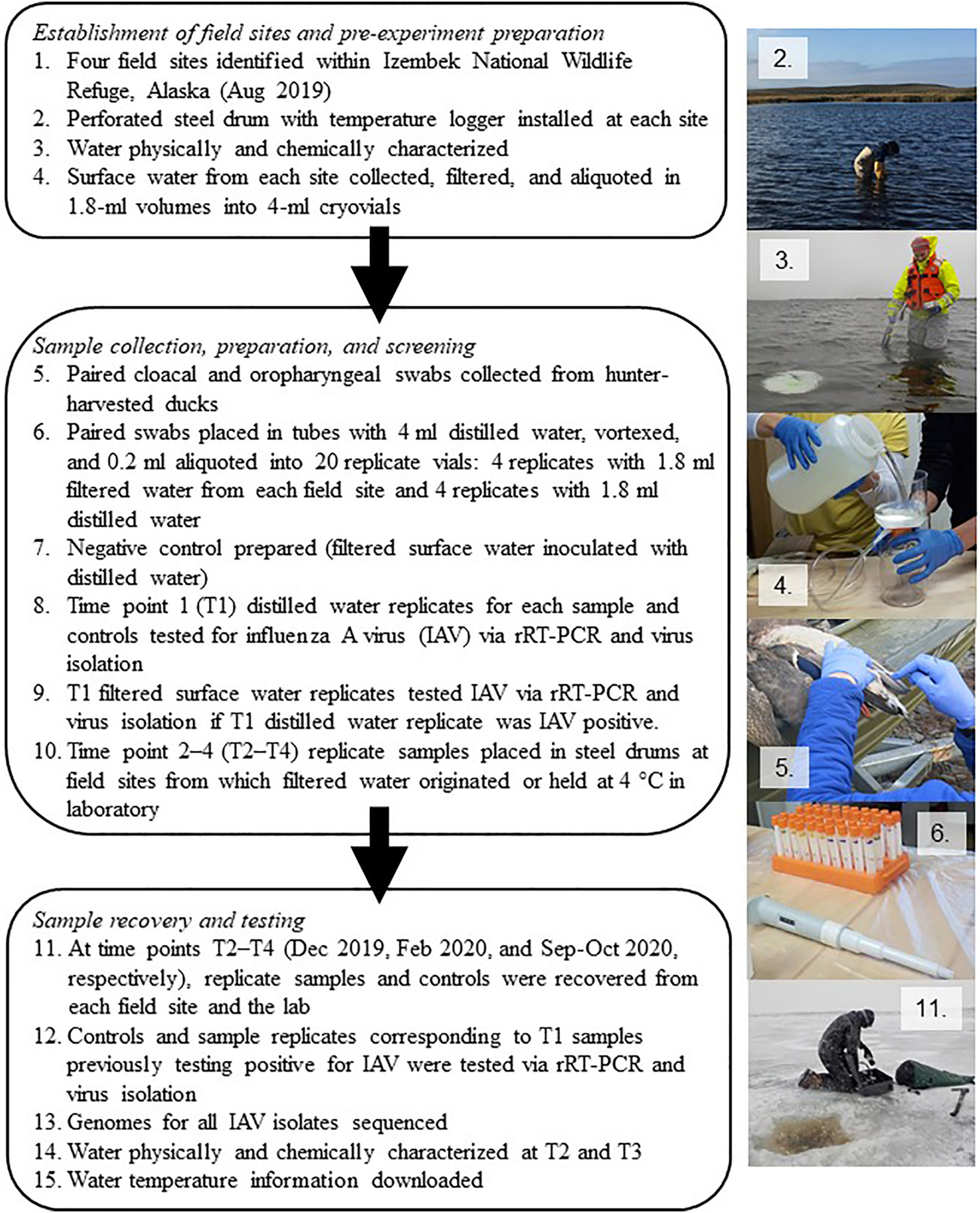
Flow chart (left) and photos (right) providing an overview of experimental field components. Polygons relate to Sections 2.1 (steps 1–4), 2.2 (steps 5–10), and 2.3 (step 11–15) of the main text. Photos depict steps 2–6 and 11 from the flowchart.

To assess the duration that avian-origin IAVs remain viable in wetland surface waters exhibiting a range of physical and chemical properties when maintained under ambient temperatures, we set up an experiment at four sites within Izembek NWR, Alaska (Figs. 1–2): Bluebill Lake, 55.25°N, 162.81°W; Proxy Pond, 55.27°N, 162.86°W; Red Salmon Lake, 55.28°N, 162.78°W; and Rescue Lake, 55.25°N, 162.83°W. Briefly, surface water was collected from four field sites and filtered to 0.22 μm. Filtered water from each field site was aliquoted in 1.8-ml volumes into 4-ml cryovials. Cryovials containing 1.8 ml of distilled water were also prepared. A steel perforated drum, equipped with a temperature logger, was submerged within each field site at a depth of approximately 1 m or less. Additionally, unfiltered water from field sites was physically and chemically characterized as follows. Standard water chemistry measures (pH, temperature, specific conductance, dissolved oxygen, and turbidity) were assessed in situ using a multiparameter water quality sonde (Yellow Springs Instruments; Yellow Springs, OH, USA) for each of our four sites. Approximately 4 l of water was also collected from each field site prior to the experiment (timepointzero or T0 henceforth; 5–7 September 2019) and sent to the lab to further characterize chemical concentrations of surface waters (inorganic anions, metals and trace elements, total dissolved nitrogen, dissolved organic carbon, and alkalinity; Table S1) using standard techniques (Aiken, 1992; Barringer and Johnsson, 1996; Brinton et al., 1996; Garside, 1982; Repert et al., 2014; Smith et al., 2006; Smith et al., 2019; U.S. Environmental Protection Agency, 1993; U.S. Environmental Protection Agency, 1994). Quality assurance and quality control included the collection and analysis of three field blanks.

### 2.2. Sample collection, preparation, and screening

Paired cloacal and oropharyngeal (CL/OP) swabs were collected from 206 hunter-harvested wild ducks. Paired swab samples from a single duck were immersed into 4 ml chilled, distilled water contained within an 8-ml screwcap tube. Contents were vortexed, and 0.2 ml were then aliquoted into each of 20 cryovials: four 4-ml cryovials containing 1.8 ml of 0.22-micron filtered surface water from each of the four field sites and four cryovials containing 1.8 ml of distilled water. Negative controls were incorporated by aliquoting 0.2 ml of distilled water into additional cryovials containing 1.8 ml of 0.22-micron filtered surface water from each of four sites or distilled water. One replicate cryovial per paired CL/OP swab sample and water type and one set of negative controls were sent to the lab upon collection (time point one = 6 September–1 October 2019; T1 henceforth) for IAV screening by real-time reverse transcriptase polymerase chain reaction (rRT-PCR) where a cycle threshold (Ct) value < 45 was considered ‘positive’ (Spackman et al., 2002) and via virus isolation (VI) in embryonating chicken eggs (Stallknecht et al., 1990b). Replicate samples consisting of distilled water with paired swab inoculum were screened first, and only sample replicates comprised of filtered surface water and inoculum corresponding to VI-positive distilled water T1 replicates identified through this initial screening procedure were subsequently tested for IAVs via rRT-PCR and VI. Prior to or concurrent with the initial testing of T1 replicate samples for IAVs, three additional replicates per paired CL/OP swab sample and water type were submerged within each steel perforated drum at each of four field sites from which filtered surface water was originally obtained, or in water held at approximately 4 °C in the lab in the case of distilled water replicate samples. All replicates were held at 4 °C upon collection (T1) prior to being placed in steel drums in the field 1–14 days later. Replicate samples remained in cryovials throughout the duration of the field/lab experiment (i.e., physically isolated from surface waters within the environment).

### 2.3. Sample recovery and testing

At three subsequent time points during the ensuing year, submersed replicate vials and controls were retrieved from the field and sent to the lab chilled on ice packs (or recovered from the refrigerator for lab-held replicates in distilled water) for IAV testing via rRT-PCR and VI. Sample collection periods for time point two (T2), time point three (T3) and time point four (T4) were 4–12 December 2019, 20–27 February 2020, and 10–16 September 2020 (for replicates held in the field) or 8–16 October 2020 (for replicates held in the lab), respectively. Only replicate samples corresponding to those previously identified as VI positive through initial screening (of T1 replicates) and negative controls were tested. The physical and chemical attributes of unfiltered environmental surface waters from field sites were also measured in situ and through laboratory analyses at T2 and T3 as previously described for T0 (including the collection and analysis of field blanks); however, we were unable to collect measures for surface waters of field sites at T4 due to logistical constraints.

To confirm results of VI and to identify any potential artifacts resulting from sampling handling or processing procedures, we genomically characterized viral isolates and assessed genetic similarity among corresponding replicate samples. To do so, RNA was first extracted from amnioallantoic egg fluids for all putative VI-positive samples using the Qiagen viral RNA mini kit (Qiagen Inc.; Germantown, MD, USA). Complementary DNA was synthesized and subsequently amplified, visualized, and purified per previously reported methods (Reeves et al., 2018). Genomic sequences for IAVs were obtained by using Nextera XT DNA Library Preparation Kits (Illumina, Inc.; San Diego, CA, USA), pooling indexed libraries, and sequencing on an Illumina MiSeq using either 500 or 600-cycle reagent kits with pairedend reads. Reads were assembled using a customized workflow on Geneious R11 (Biomatters Ltd.; Auckland, New Zealand) using reference data for IAVs obtained from GenBank (Clark et al., 2016).

### 2.4. Analysis and interpretation

Genomes of IAVs obtained from all T1 replicates derived from the same paired CL/OP swab sample were compared, and only those samples sharing >99% identity among all replicates (minimum of two) were used to assess persistence through time (i.e., to avoid deriving inference from field or laboratory artifacts which could result from heterogeneous replicates of samples representing mixed IAV infections, mutations induced through egg culture, or cross contamination of samples). Genomes of IAVs recovered from VI-positive T2, T3, and T4 replicate samples that shared >99% nucleotide identity at all gene segments with each other and corresponding viruses recovered from the T1 VI-positive distilled water replicate samples were inferred to remain infectious at the time point at which samples were retrieved. In the case of isolates representing mixed infections, we considered T2–T4 replicates to remain infectious if nucleotide sequences for either or both of two alleles for a given gene segment shared >99% nucleotide identify with corresponding gene segment sequences generated for the T1 distilled water isolate. T2–T4 replicates yielding IAV isolates with genomes sharing <99% nucleotide similarity at any gene segment as compared to the T1 distilled water isolate were considered negative to avoid deriving inference from field or laboratory artifacts as described above.

To assess potential differences in viral persistence among replicates held in the field at four wetland sites and in the laboratory, we applied a Kaplan-Meier survival analysis to IAVs assessed for viral infectivity through time. To estimate the survival interval for viruses maintained under laboratory and field conditions, we considered T1 to be our start date which varied among samples. We considered the survival endpoint for any given replicate to be the mean date between the last time point from which we recovered an infectious IAV and the next subsequent time point at which the corresponding replicate was collected and tested negative (i.e., the time point from which no further infectious IAV was recovered for a given replicate sample). Survival curves and confidence intervals were calculated using the survival package (Therneau, 2020) in R (R Core Team, 2021).

All data supporting conclusions in this product have been made publicly available in an associated data release (Reeves et al., 2021) and via GenBank (accession numbers MW874296–MW874409).

## 3. Results

### 3.1. Initial sample screening

**Fig.3.**
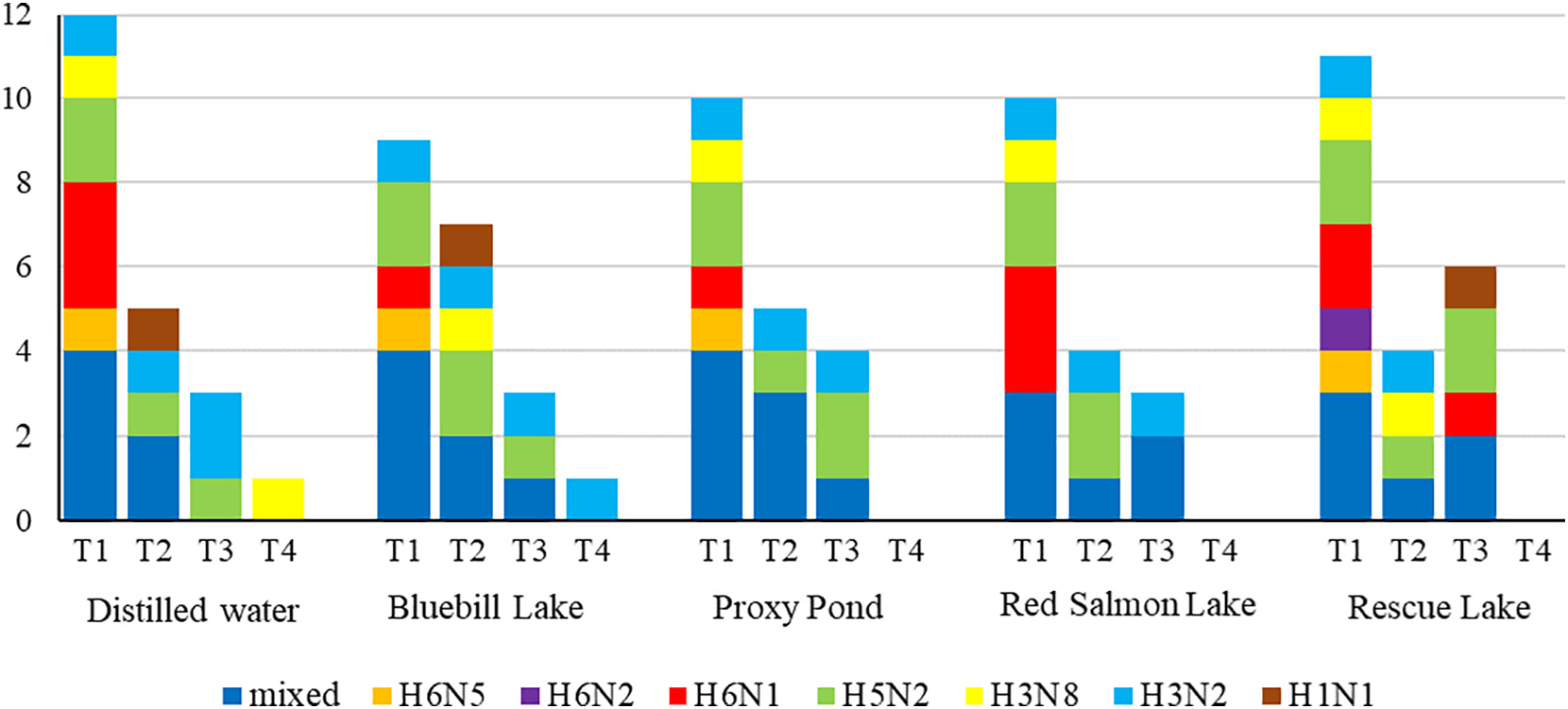
Number of sample replicates determined to contain infectious influenza A viruses *in ovo* upon retrieval at four time points (T1 = 6 September–1 October 2019; T2 = 4–12 December 2019; T3 = 20–27 February 2020; T4 = 10–16 September 2020 for samples maintained at field sites and 8–16 October 2020 for replicates held in the lab) as confirmed through genomic characterization and comparison. Subtype combinations for viruses recovered are shown for each time point. Replicates were held at 4 °C in the laboratory (distilled water) or at ambient environmental temperatures at four sites within Izembek National Wildlife Refuge, Alaska (Bluebill, Lake, Proxy Pond, Red Salmon Lake, and Rescue Lake).

From the initial (T1) screening of 206 paired CL/OP swabs from ducks aliquoted into distilled water, we identified IAV RNA in 58 samples (28.2%) via rRT-PCR and infectious IAV in 15 samples (7.3%) through VI. Through the testing of the T1paired swab samples aliquoted in filtered surface water from each of four Alaska study sites that corresponded to the 15 VI positive samples, and subsequent genomic characterization of resultant isolates, we identified 12 samples that met our criteria for assessing the duration of viral infectivity (Fig. 3; Supplemental Tables S2–S113). Three samples were omitted from downstream analyses given a lack of isolation of IAVs from nondistilled water T1 replicates (sample 181) or the finding of IAV genomes that did not exhibit >99% similarity across all corresponding gene segments for replicate samples (samples 163 and 190; Supplemental Tables S50, S83, S85, and S87) which may have resulted from field or laboratory artifacts (e.g., heterogeneously mixed replicate samples, mutations induced by egg culture, or cross contamination of samples). Combined subtypes represented by the 12 IAVs for which persistence was assessed through time included: H1N1, H3N2, H3N8, H5N2, H6N1, H6N2, H6N5 as well as numerous mixed infections (Fig. 3). All T1 negative controls were negative for IAV via both rRT-PCR and VI.

### 3.2. Testing of samples recovered through time

Through the testing of T2, T3, and T4 replicate samples for each of five treatments (distilled water held in the lab and filtered surface water from each of four Alaska study sites and held in the field), we observed successive declines in infectivity through time in the number of replicates testing VI-positive for viable IAVs per treatment with the single exception of T3 replicates held within, and containing surface waters from, Rescue Lake (Fig. 3). Six T3 replicates held in Rescue Lake samples were IAV positive via VI in contrast to four T2 replicates maintained within this same site. In several instances (samples 99, 160, and 174), culture of T2–T4 replicates maintained in distilled or filtered surface water yielded pure culture of a single IAV whereas the initial T1 distilled water had included a mixed infection. At least three IAVs (≥25%) remained infectious in replicates for each treatment type through T3 (i.e., isolates were recovered from samples retrieved from the field or lab for testing 144–155 days since initial sample collection). Only two T4 replicate samples yielded infectious IAV when retrieved and tested after a period of 361–377 days: one distilled water replicate (for sample 160) which had been held in the lab at approximately 4 °C and one replicate (for sample 99) containing surface water from Bluebill Lake and maintained under naturally occurring temperatures at this site within Izembek NWR, Alaska (Fig. 3). The IAVs isolated from T4 replicates containing distilled water and surface water from Bluebill Lake were apparent pure cultures of the H3N8 and H3N2 IAVs, respectively, though corresponding T1 replicates of these samples represented mixed infections (Fig. 3). No IAVs were recovered at times T2–T4 from negative controls. One isolate from a T2 distilled water replicate (for sample 198) shared <99% identity at the NP gene segment as compared to the isolate from the corresponding T1 replicate, potentially an artifact of egg culture; therefore, we considered this T2 replicate IAV negative per our a priori criteria (see Materials and methods).

### 3.3. Survival analysis

**Fig. 4.**
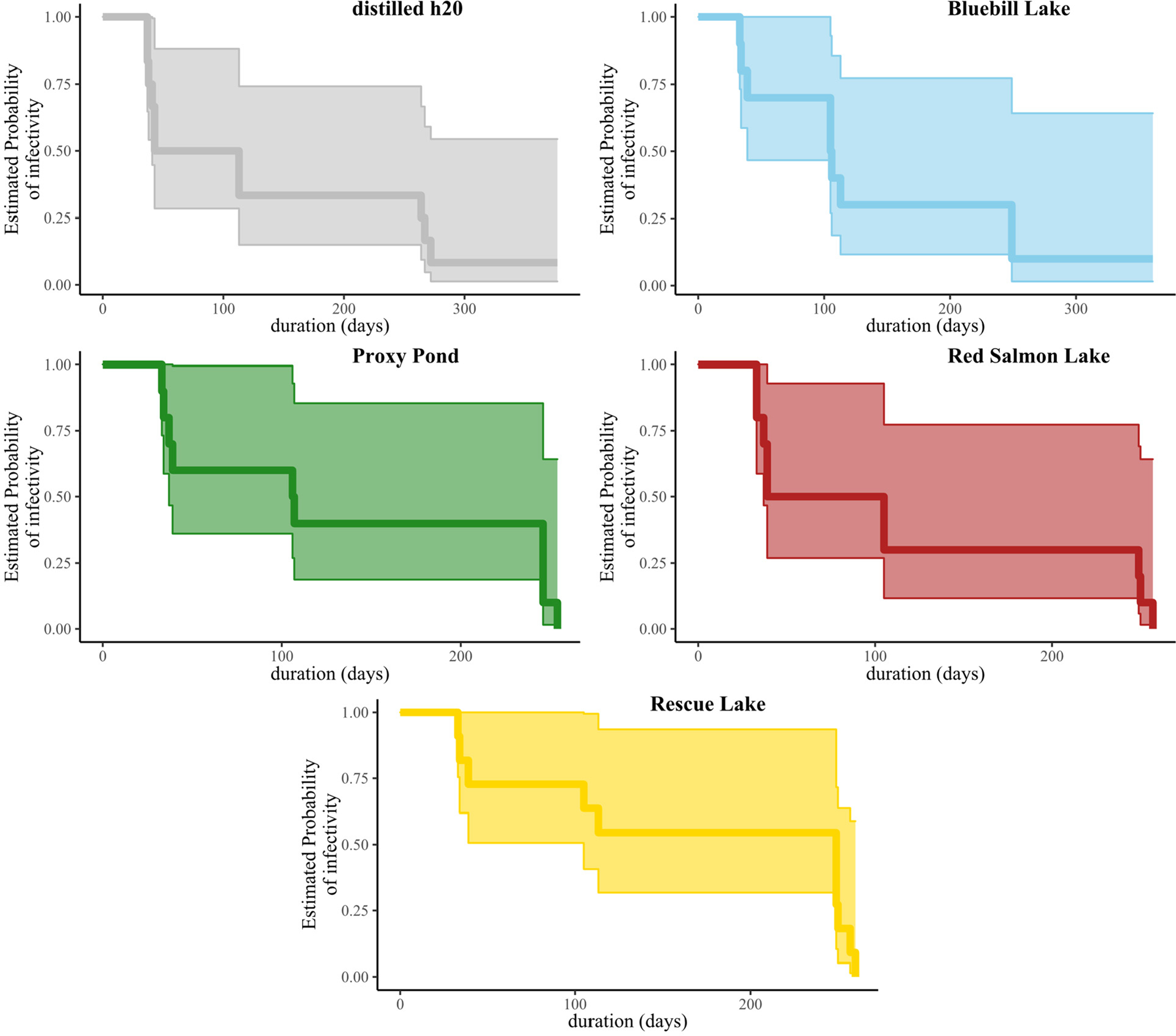
Kaplan-Meier survival curves depicting the estimated probability of infectivity for influenza A viruses through time when held at 4 °C in laboratory (distilled water) or at ambient environmental temperatures at four sites within Izembek National Wildlife Refuge, Alaska (Bluebill, Lake, Proxy Pond, Red Salmon Lake, and Rescue Lake). Shaded areas represent 95% confidence intervals for each curve.

Our Kaplan-Meier survival analysis provided evidence for decreasing probability of infectivity through time for IAVs maintained under five different treatments (Fig. 4). Estimates of infectivity for IAVs maintained under all treatments remained within the second and third probability quartiles (0.75–0.25) from day 50 through day 200 with considerable overlap of confidence intervals among treatments (Fig. 4). The estimated probability of infectivity reached zero for Proxy Pond, Red Salmon Lake, and Rescue Lake replicates prior to or on day 260 whereas the estimated probability of infectivity remained at ≥8% for distilled water and Bluebill Lake replicates through day 377 and 361, respectively (Fig. 4).

### 3.4. Physical and chemical characterization of surface waters

Water temperatures at four Izembek NWR field sites within which replicate samples were held were generally cool and consistent among sites with mean daily temperatures ranging between 0.0 and 18.7 °C (Supplemental Fig. S1). The overall mean daily temperature throughout the 343- to 349-day period during which replicate samples were held ranged between 7.2 and 7.6 °C (Supplemental Fig. S1). Measures of surface water pH at these same four sites at three time points were near neutral, ranging from 6.15 to 8.01 (Supplemental Fig. S2). Additional in situ measures of surface water properties and subsequent chemical analyses of water samples collected from each of four sites at three time points provided measures at or below the reporting limit (Supplemental Figs. S3–S15), slightly greater than the reporting limit (rubidium, tungsten, and vanadium; Supplemental Figs. S16–S18), consistently higher at Proxy Pond as compared to other sites (specific conductance and concentrations of aluminum, boron, bromine, chlorine, dissolved organic carbon, iron, magnesium, manganese, potassium, sodium, strontium, sulfate, total dissolved nitrogen; Supplemental Figs. S19–32), or lacking clear trends within and among sites (Supplemental Figs. S33–40). Two inorganic analytes (nitrate+nitrite-nitrogen and nitrate) were detected in two field blanks and therefore were not reported (Supplemental Table S114). Given small volumes and heterogeneity among samples, we did not characterize physical and chemical properties of contents for individual replicate samples held within, but physically isolated from, surface waters at each field site.

## 4. Discussion

In this investigation, we found evidence that IAVs from naturally infected ducks may remain infectious for extended periods of up to approximately one year, and potentially longer, when maintained in filtered surface water under ambient temperatures in southwestern Alaska wetlands at Izembek NWR or in temperature controlled distilled water in the laboratory. None of our negative controls containing filtered water from field sites inoculated with distilled water were identified as IAV-positive via rRT-PCR or VI, providing evidence that surface water used in this investigation was IAV-negative. Therefore, we have confidence that inference on the duration of infectivity was derived from IAVs present in CL/OP swabs from ducks. This study also provides field data that IAVs may commonly remain infectious for periods of months after deposition when maintained in water with cool temperature, near-neutral pH, and exhibiting relatively low to moderate specific conductivity, corroborating results reported for previous laboratory-based investigations (Keeler et al., 2014). As such, results of our study both corroborate and extend the body of evidence that environmental surface waters may represent an important medium in which infectious IAVs may be maintained for extended periods outside of a biotic reservoir.

The finding that one IAV, maintained in filtered surface water from Bluebill Lake and held under ambient temperatures at this field site overwinter, remained infectious after approximately one full year extends the maximum duration for which viral infectivity has been confirmed through field experimentation by approximately five months (Ramey et al., 2020). This result also exemplifies that the durational limits for the environmental persistence of infectious IAVs in wetland surface waters under ecologically relevant conditions have not yet been fully characterized. As environmental persistence has been posited to play an important role in recurrent wild bird epidemics (Breban et al., 2009), additional information on the duration of IAV persistence in North American surface waters may be biologically relevant towards accurately identifying the dynamics of future outbreaks in wild birds.

Though we did not estimate viral titers for duck swabs in filtered surface waters and distilled water at T1 in our experiment, which likely varied among samples and influenced the duration of infectivity, the finding that >25% of IAVs present on swabs collected from naturally infected ducks remained infectious in each water type under ambient temperature conditions for >144 days, or the equivalent of nearly five months, suggests that a considerable proportion of wild bird origin IAVs have the capacity to persist in diverse wetland surface waters for long durations, and potentially over winter, at high latitude locations. We recognize that viral strain, viral titer, water chemistry, and numerous other factors likely influenced temporal viral viability in our study. Solar ultraviolet radiation and biotic components of surface water are other factors that likely affect the viability of IAVs, though we attempted to control for these influences in our investigation. That is, steel perforated drums were sealed on the ends to minimize the entry of ambient light and filtering of surface waters was intended to minimize algae, bacteria, biofilms, invertebrates, and other biologics that could have inhibited, or in some cases promoted, the duration of viral infectivity.

In this study, we attempted to incorporate a diversity of field sites and temperature regimes to provide inference on variability of viral persistence in surface waters with diverse chemical properties at a local scale. Surface water from three of our sites consistently shared similar physical and chemical properties (Bluebill Lake, Red Salmon Lake, and Rescue Lake) while only one site showed evidence for marked differences in chemical attributes (Proxy Pond), likely a function of tidal influence to this latter site. As such, we acknowledge that the water in which IAVs were maintained in this study may have represented a relatively narrow diversity of physical and chemical properties. We also recognize that our measures of surface waters at field sites do not necessarily reflect the same physical and chemical attributes of sample replicates (in cryovials) physically isolated from the environment throughout our experiment. Thus, we were unable to draw any rigorous inference from this study regarding how variability of physical and chemical properties of surface water among field sites influenced the duration of viral infectivity.

Additional evaluations incorporating ecologically relevant context are necessary to better understand the viability of IAVs in globally diverse wetlands. Such evaluations are particularly important for high consequence IAVs such as highly pathogenic clade 2.3.4.4 Goose/Guangdong lineage H5 viruses. By better understanding the persistence of IAVs in diverse environmental surface waters, agencies managing wild water birds and their aquatic habitats may be able to better mitigate damages from outbreaks of highly pathogenic avian influenza involving wild birds through the development of science-based management actions. For example, agencies could explore: (i) limiting access to potentially virally contaminated wetlands for a defined period following outbreak events to avoid/reduce spread via fomites; (ii) regulating the disinfection of boats, hunting/fishing gear, and/or equipment following use in potentially virally contaminated wetlands for a defined period following outbreak events; (iii) instituting or increasing surveillance effort for high consequence IAVs in wild birds or wetlands affected by outbreaks for a defined period following detection events; and (iv) managing water levels or altering the pH/temperature of small ponds affected by outbreak events to facilitate more rapid viral deactivation and/or to mitigate further viral dissemination via water. Ideally, the potential development and implementation of management activities aimed at mitigating viral dissemination via water would incorporate ecologically relevant information on the duration of IAV infectivity as well as empirical evaluation of how infectivity may be altered or mitigated for through science-based actions.

## 5. Conclusions

Through a combination of laboratory and field-based experimental approaches, we assessed the persistence of 12 IAVs obtained from the cloaca and/or oropharynx of naturally infected ducks in filtered surface water over approximately one year. Our results provide evidence that most IAVs remained viable for extended periods that may exceed one year when maintained in cool water, with near-neutral pH, and exhibiting relatively low to moderate salinity/specific conductivity. Therefore, wetlands exhibiting these conditions may represent an important medium in which infectious IAVs may reside outside of a biotic reservoir.

## Supplementary Material

Supp Figure S1

Supp Figures

Supp Tables

## HIGHLIGHTS

- We assessed the persistence of 12 influenza A viruses in lake water over one year.
- Influenza A viruses remained viable in surface water for extended periods.
- Some influenza A viruses remained viable beyond one year.
- Wetlands represent a medium in which infectious influenza A viruses may reside.

## Abbreviations

C: Celsius
CL/OP: Cloacal and oropharyngeal
Ct: Cycle threshold
DNA: Deoxyribonucleic acid
IAVs: Influenza A viruses
l: Liter
ml: Milliliter
N: North
NP: Nucleoprotein
NWR: National Wildlife Refuge
RNA: Ribonucleic acid
rRT-PCR: Reverse transcriptase real-time polymerase chain reaction
T0: Time period 0, equivalent to 5–7 September 2019
T1: Time period 1, equivalent to 6 September–1 October 2019
T2: Time period 2, equivalent to 4–12 December 2019
T3: Time period 3, equivalent to 20–27 February 2020
T4: Time period 4, equivalent to 10–16 September 2020 for replicates held in the field or 8–16 October 2020 for replicates held in the lab
VI: Virus isolation
W: West
