# Supplementary figures and images for: Evidence for interannual persistence of infectious influenza A viruses in Alaska wetlands
Source: Sci Total Environ. Author manuscript; Available in PMC 2022 Jul 13. (PMC9277558; doi:10.1016/j.scitotenv.2021.150078)

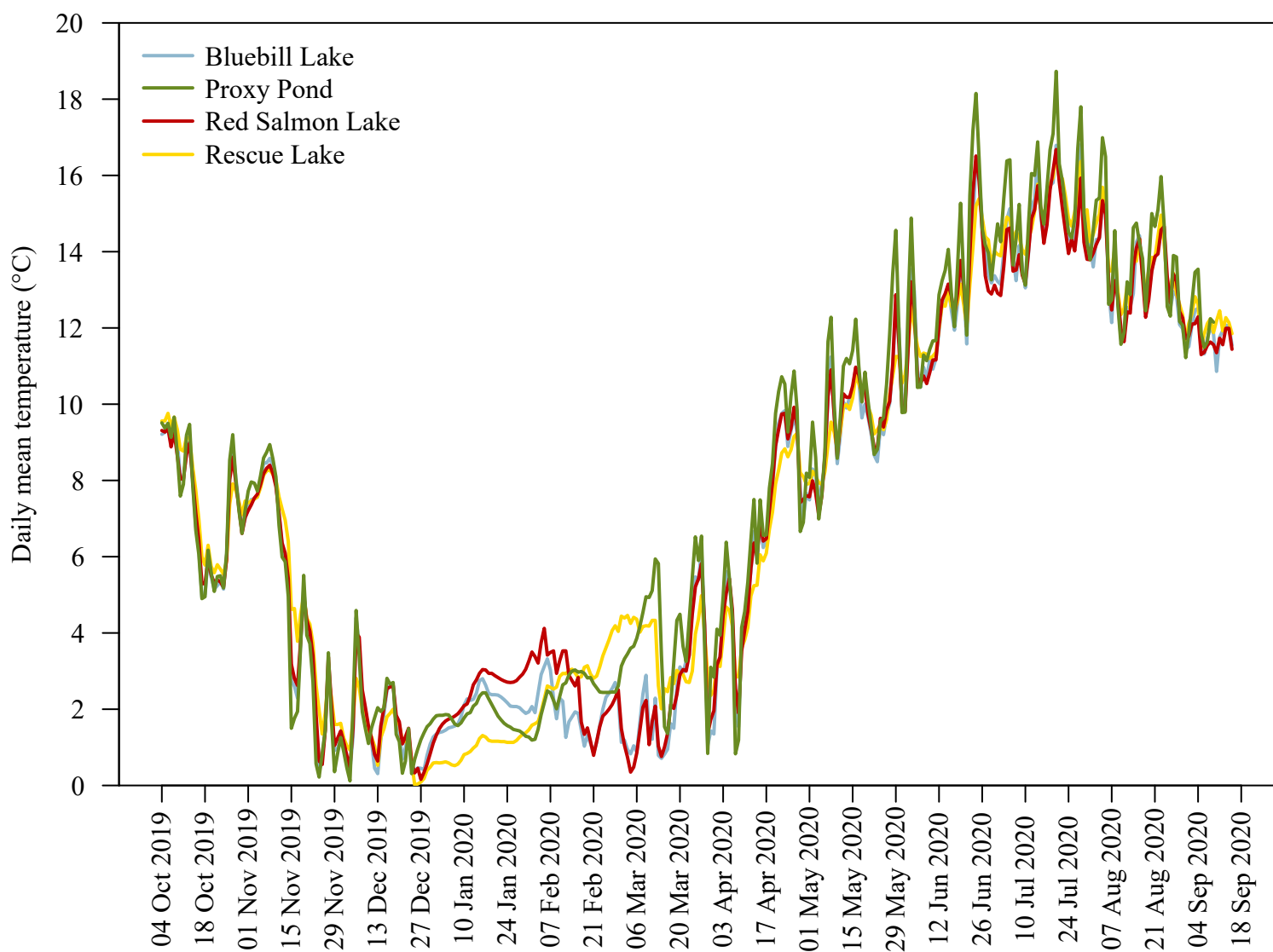

Supplement: Supp Figure S1 [file NIHMS1818455-supplement-Supp_Figure_S1.pdf]
